# The Science of Hygiene

**Published:** 1890-10

**Authors:** John Sheppman


### THE SCIENCE OF HYGIENE, AND ITS CORRUPTIONS.

BY DR. JOHN SHEPPMAN.

The great question—"How to prolong life," with its numerous detailed advises, has, no doubt, become monotonous to the general public. The fundamental theories adopted by eminent physicians have proved themselves so contradicting, as to make most of our intelligent men regard their doctrines with more or less ridicule. There is no subject more universally assailed, and corrupted, than this great science of hygiene.

Reviewing the combined issue of one of our reputable health journals, I have come to the conclusion that it is absolutely impossible for us to live profoundly in accordance with the rules and regulations assigned to us by our distinguished medical authorities. The pabulum of life has been seriously besieged. The atmosphere, our vital air, has been microscopically discovered to be filled with rank and poisonous disease germs. And now, since we are aware of the deplorable state into which we are unceremoniously placed, it only remains for some generous writer to inform us that we had better close up our accounts and quit eating, and likewise breathing.

What is hygiene? And how should we live? The most correct answer to this is to follow the advice of the great Pythian of old, who has said:—"Know thyself." Study the conditions of your own constitution, and endeavor to live in close harmony with the established, and respective laws of nature.

That disease *is* the result of our own negligence, has, I believe, been definitely shown us. And that many lives are annually lost through sheer ignorance of this subject, must also be admitted. Now, taking into consideration these clear important facts, it surely remains for us to ask ourselves how much do we know about this great science? Are we not cruelly neglecting the health of ourselves, and fellow creatures? And do we not suffer many ills which might by some simple method be effectually remedied? Let us pause for a moment upon the probable causes of our "National malady"—dyspepsia. We may all know very well that the etiology of this disease can be directly traced to diet. And that improper food with its manifold objections forms the basis for more bodily ills, than all the outward influences combined. For, with the recognized germ theory, are we not obliged to believe that the susceptibility of the system to disease depends chiefly upon the weak, or the vigorous state of the body? And do we not owe our health, our strength, our blood, ay, yes, our very lives, to the wonderful machinery of the stomach? But, what are the influences which are intimately associated with the formation of this dreadful disease? Why, I think that we can truthfully say, the brain, the feet, the nerves, and every petty fibre plays an important part in preserving, or destroying, the natural function of digestion.

Each man is a law unto himself, but there are many laws which must be observed by every individual.

The awful ravages which disease makes upon mankind ought to be an inspiration for every man to arm himself cap-a-pie, and to institute an indubitable search into every suspicious source of virulent contagion. How much we need the teaching of hygiene and physiology in our public schools was illustrated to me by a remark made by a prominent member of the bar of one of our great western cities, signifying that he cannot see how the head, and the feet, can conveniently affect the stomach. This gentleman will undoubtedly listen to, believe, and retell some of the weird and fanatical tales which were told immediately after the massacre of "St. Bartholomew," relating to the awful butchery, describing acephalous forms parading the streets with their own heads under their arms, while others were vainly endeavoring to pick from the gory piles, some skulls particularly suitable to their peculiar tastes.

Oh, let us preach hygiene, with pomp and eloquence, for, while the pulpit prepares for the future of the soul, why should we not with equal zeal, endeavor to improve the conditions of its earthly residence.

Descartes, the great geometrician, has said :—"That as for rendering man immortal, it is what I will not venture to promise; but I am very sure that I can prolong his life to the standard of the Patriarchs." This may be true. But let us remember that with all these proclamations, the greatest work is done by one grand combine, an indomitable effort to educate the masses how to prevent a majority of the 899 diseases,* which are weekly, daily, hourly, wreaking their vengeance upon suffering humanity.

Punishment holds full reign; every violation is followed with a natural pang. To create legislative laws for the enforcement of sanitary measures, would, at first, appear tyrannical to a majority of the people, and probably lead to a general revolt, which would re-act in accordance

*According to a report issued by the Royal College of Physicians, London, Eng.

with the degree of intelligence possessed by the people, and the amount of literature bearing upon hygiene and its precautions, judiciously, and illustratively disseminated in their midst.

The greatest charm of life is health, and with health alone can beatitude make equal strides. We do not hold, advocate, nor declare that each man should live in constant dread of impending disease! Nor that any one should imagine himself afflicted with a score of bodily ills! But, that we should all look upon life in an intelligent, mechanical, and spiritual way, and recognize the prime and simple causes of many of our distressful maladies, for, I think that we can truthfully say that most of our people do not understand the detrimental influence of what they eat and drink, and that we do not permit our brains and limbs to have the requisite amount of nature's mysterious recuperating process—Sleep.

Oh, man of fortune, what is life
If in it thou dost seek but wealth;
And with thy maddening endless strife
Dost heedlessly ignore thy health?

Once more let me impress upon you the direct relationship existing between rest and a sound mind, and a higher and more perfect physical development. So then, when the glittering lights, the whirling dance, the festive board, the bubbling glass, and all the midnight gaieties close alluringly around us, oh, that we all could stand with a firm resolve, and like the Frenchman, say, "Mon lit me demande;" my bed demands me. For, it is there that our limbs find strength and comfort; where the ills of life are nursed with knowledge; where the troubled heart finds sweet repose. And it is there I hope that we all will lie at the twilight of life's ending journey, when the soul shall be borne from the pier of the world, into the glorified bliss—Eternal.

